# Image Processing and Deep Convolutional Neural Network Method for Automated Malaria Parasite Detection in Thin Blood Slide Images

**DOI:** 10.3390/diagnostics16132091

**Published:** 2026-07-03

**Authors:** Kavita Kumari, Taruna Kaura, Abhishek Mewara, Suman Tewary, Neerja Mittal Garg

**Affiliations:** 1 Academy of Scientific and Innovative Research (AcSIR), Ghaziabad 201002, India; kavita.csio19j@acsir.res.in; 2 CSIR-Central Scientific Instruments Organisation (CSIR-CSIO), Sector 30-C, Chandigarh 160030, India; 3 Department of Medical Parasitology, Postgraduate Institute of Medical Education and Research, Sector 12, Chandigarh 160012, India; kaurataruna@gmail.com (T.K.); abhishekmewara@gmail.com (A.M.); 4 Materials Engineering Division, CSIR-National Metallurgical Laboratory (CSIR-NML), Jamshedpur 831007, India; sumantewary.nml@csir.res.in

**Keywords:** convolutional neural network, computer-aided diagnosis, densely connected convolutional network, polymerase chain reaction, rapid diagnostic test, visual geometry group network

## Abstract

**Background:** Malaria is a life-threatening disease caused by *Plasmodium* species, which is endemic in tropical and subtropical regions worldwide. In clinical settings, experienced parasitologists perform microscopic examinations of thick/thin blood slides. This method is labour-intensive and is adversely affected by inter- and intra-observer variability among the microscopists. The present study aimed to develop a malaria screening algorithm using computer vision to identify and classify malaria parasite-infected red blood cells (RBC) from microscopic blood slide images. **Methods:** The proposed classification methodology first employs digital image processing techniques, the watershed transform, to preprocess the raw images, followed by connected component labelling to accurately segment and isolate individual RBCs from the background. To classify these segmented cells as either normal or infected, convolutional neural networks (CNNs) were utilized, leveraging their ability to automatically extract relevant features through deep, hidden layers, thus eliminating the need for manual feature engineering. **Results:** To compare and determine the most effective classification engine, the study developed and evaluated five distinct models: four well-established transfer learning architectures (*VGG16*, *VGG19*, *DenseNet121*, and *InceptionV3*), alongside a newly proposed custom CNN model. A total of 2422 segmented RBC images were used for the training, and 692 different images were used for testing, with the *VGG* model showing the best accuracy at 99.57%. The proposed CNN architecture also showed competitive results with 99.14% accuracy. **Conclusions:** Transfer learning models demonstrated remarkable accuracy for malaria parasite classification from blood smear slides, with VGG19 (99.57%) achieving the highest accuracy on diverged datasets for the test images. The analysis demonstrates the potential of this approach as a computational aid for future image-based malaria screening in conjunction with existing diagnostic tests.

## 1. Introduction

Malaria is a potentially life-threatening disease caused by protozoan parasites of the genus *Plasmodium*, which infect and proliferate within the human red blood cells (RBCs). The transmission of malaria occurs via the bite of infected female *Anopheles* mosquitoes, which serve as biological vectors for the parasite. Multiple species, including *Plasmodium falciparum*, *P. vivax*, *P. malariae*, *P. ovale*, and *P. knowlesi*, can infect humans. The level of malaria parasitemia (MP) is assessed by quantifying the RBCs infected by *Plasmodium* parasites, which helps determine the severity of the infection [1]. The most effective approach to preventing malaria is early diagnosis and prompt treatment. In 2023, there were 263 million cases and 597,000 deaths worldwide, as reported by the World Health Organization [2]. Children under five years old are the most vulnerable group, comprising 61% of estimated malaria-related deaths. Thick blood smears are utilized to detect the presence of *Plasmodium* parasites, offering enhanced sensitivity for parasite detection compared to thin smears. In contrast, thin blood slides are utilized to identify the specific species and life-cycle stage and to compute parasitemia. Alternative techniques are available, such as polymerase chain reaction (PCR)-based tests, which are expensive for wide use in disease-endemic regions, and rapid diagnostic tests (RDTs), which have limited sensitivity [3,4,5]. Researchers have developed several computer-aided diagnosis (CAD) software programs for identifying malaria-infected cells from slide images. Advancements in computer vision approaches for malaria diagnosis leverage image processing, machine learning (ML), and deep learning (DL) methods [6,7]. The CAD image analysis software employs ML methods that utilize hand-crafted features for diagnosing malaria from slide images [6,7,8]. The classification of malaria parasites is done in two stages using ML techniques. The initial stage classifies cells as either normal or infected by analyzing texture and color features. The subsequent stage incorporates additional attributes, including shape, relative size, parasite color, as well as the number, size, and positions of parasites within the cell, to distinguish parasite species (*falciparum*, *vivax*, *malariae*, or *ovale*). A normalized RGB color space [9] technique is used for pixel classification to differentiate between RBCs and the background. Subsequently, an inclusion-tree representation is used to identify the RBCs. A support vector machine (SVM) employing a polynomial kernel is implemented to classify RBCs as either infected or normal. A detection approach has been proposed [10] that uses thresholding and stage classification (schizont and trophozoite) based on 23 morphological features extracted from unstained slides. This method applies k-nearest neighbour (KNN), logistic regression (LR), and linear discriminant analysis (LDA) classifiers. Another method focuses on separating overlapping cells [11] by utilizing morphological operations and analyzing focal points in concave regions to enhance RBC quantification. Additionally, it extracts histogram-based features such as LBP and HOG to classify malaria parasites into different life-cycle stages, using Naïve Bayes, KNN, and multi-class SVM.

However, these ML methods with hand-crafted feature engineering for the automated malaria analysis process have limitations. They need expertise in analyzing textural and morphological features, as well as the position and angle of the image’s region of interest (ROI), and background. To overcome these challenges, data-driven DL models combine layers with non-linear processing units that can solve this problem by self-learning features in the raw data without designing any feature sets, extracting the end-to-end hierarchical features, and performing classification. DL methods have revolutionized the field of artificial intelligence and ML, promising scalable, improved performance with increased computational resources and data size [12]. The method based on CNNs has played a vital role in this revolution and has performed very well in medical image analysis.

The DL models using CNNs, a hierarchical architecture of learning, have shown promising results in image recognition, localization, and classification tasks [13,14]. The CNN models utilize spatial correlations between neighbouring voxels or pixels to capture local patterns, such as edges in an image, thereby minimizing the number of parameters that must be learned. The key features of CNNs include local connectivity, shared weights, pooling layers, and multiple layers. This improves the accuracy of the feedforward-backpropagation training procedure. CNN-based DL models, including *AlexNet* [13], *VGGNet* [15], *ResNet* [16], *GoogLeNet* [17], *Xception* [18], *Inception-V3* [19], and *DenseNet* [20], have demonstrated notable advancements in performance. These models achieve significant improvements in CNN efficiency by utilizing fewer parameters and computations for natural image classification tasks conducted as part of the *ImageNet* Large-Scale Visual Recognition Challenge (*ILSVRC*) [21].

The current literature underscores the adoption of transfer learning in medical image analysis, primarily attributed to the constrained availability of annotated datasets. A model trained on the *ImageNet* dataset is employed as generic feature extractor and fine-tuned on specific tasks on the new datasets for image recognition [22]. Current studies reveal that the application of CNN-based DL models provides promising performance in detecting malaria parasites from thin blood slide images. A comparison of kernel-based approaches [23], such as SVM, and pre-trained CNN models, i.e., *GoogLeNet*, *AlexNet*, and *LeNet*, was used to classify normal and parasitized cells. The results showed the CNN models outperformed the SVM classifier, achieving an accuracy of 95%, while the SVM achieved 92%. The authors [24] proposed a custom-designed CNN model that analyzes the focus stack of slide smear images and achieves a 98.77% Matthews Correlation Coefficient (MCC) score. In another work [25], authors used a large-scale clinical annotated dataset to find the optimal layers of a pre-trained DL model for feature extraction to discriminate between normal and parasitized cells. They also performed statistical validation of their results and observed that the pre-trained *ResNet-50* model demonstrated superior performance compared to other CNN models, achieving an accuracy of 95.9%. In a recent work [26], authors have constructed an ensemble model with *SqueezeNet* and *VGG19* to reduce model variance and improve generalization and robustness towards classifying normal and parasitized cells. The related work on malaria classification is shown in Table 1.

**Figure 1:**
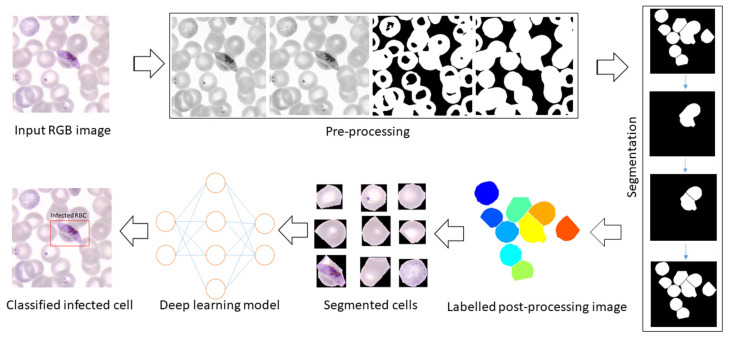
Overview of the proposed methodology for parasite classification.

This study presents a computer-assisted method to enhance automated malaria disease screening. A schematic representation of the algorithm employed is shown in Figure 1. This includes the introduction of a method for segmentation of RBCs from microscopic blood slide images, followed by their classification as healthy or infected. The predominant species, *P. falciparum* and *P. vivax*, are identified based on internal characteristics such as texture, morphology, and other features. A complete automation of RBC quantification is hindered by overlapping cells, so the present work also explored the quantification problem of clumped cells. This method distinguished overlapping shapes through the application of morphological and convexity analysis, which relies on implicit conditions related to cell size and shape and separates them using a watershed. Additionally, DL-based CNN architectures have been designed for the classification of malaria-infected cells, utilizing segmented RBCs extracted from whole images.

The primary contributions of this study include the development of architectures for various pre-trained DL models through modifications to their final fully connected (FC) layers and the creation of customized CNN models for the classification of parasitized and uninfected cells. Additionally, a comparative assessment was conducted to identify the most effective CNN architecture.

## 2. Materials and Methods

### 2.1. Sample Image Dataset

**Database 1 (DB1):** Microscopic images of Giemsa-stained thin peripheral blood slides from human blood samples infected with *Plasmodium* parasites were captured from the slides by trained microscopists using an *Olympus BX3-25ND25* digital microscope (100× magnification and ten μm pixel resolution) at the Department of Medical Parasitology, Postgraduate Institute of Medical Education and Research (PGIMER), Chandigarh. The study received approval from the Institute Ethics Committee (IEC) of PGIMER, Chandigarh (PGI/IEC/2021/001348).

**Database 2 (DB2):** This dataset has been downloaded from the online repository DPDx-Laboratory Identification of Parasites website, managed by the Division of Parasitic Diseases and Malaria (DPDM) at the CDC, Atlanta, USA (Centers for Disease Control and Prevention) [30]. The exact numbers of samples and RBCs are given in Table 2.

**Figure 2:**
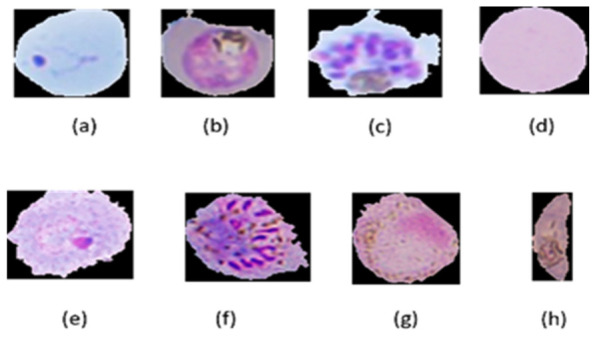
Cell Images: (**a**) PFR, (**b**) PFT, (**c**) PFS, (**d**) NOR, (**e**) PVR, (**f**) PVS, (**g**) PVG, and (**h**) PFG.

The study focused on two parasite species infected cells, which include a combination of seven life-cycle stages infected cells (PFR: *P. falciparum* ring, PFT: *P. falciparum* trophozoite, PFS: *P. falciparum* schizont, NOR: normal, PVT: *P. vivax* tropphozoite, PVS: *P. vivax* schizont, PVG: *P. vivax* gametocyte, and PFG: *P. falciparum* gametocyte). Figure 2 represents a sample of infected and normal cells, highlighting the target cells for both the datasets (DB1 and DB2).

### 2.2. Segmentation of RBCs

The proposed pipeline leverages standard image enhancement techniques followed by a watershed to accurately delineate cell boundaries. A two-stage distance transform watershed is applied for accurate segmentation and to overcome the overlapping cells segmentation, as well as the over-segmentation of the cells, from complete microscopic images.

**Figure 3:**
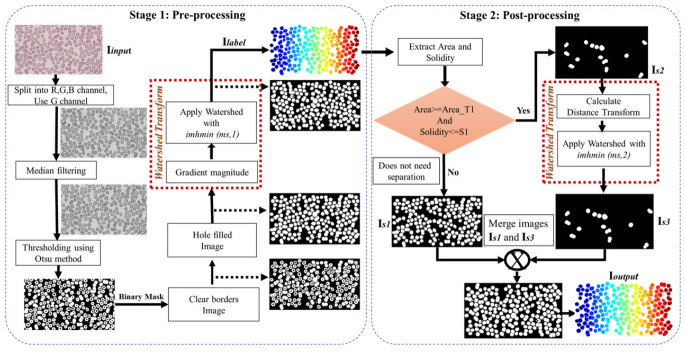
Flowchart of the distance transform watershed algorithm.

The first stage addresses the issue of over-segmentation (Figure 3). An h-min value of 1 is applied along with the distance transform watershed to produce the initial segmented image. However, some of the overlapping RBCs remain unsplit, i.e., under-segmentation (Figure 3). In the second stage, clusters within the remaining overlapping cells of the image are identified based on key characteristics of the RBCs, such as their convexity and size. For each enclosed region, the solidity value is defined as follows:

(1) $$Solidity=\frac{Area}{Convex\;Area}$$

where Area denotes the area of the closed region, and Convex Area refers to the area of the smallest convex polygon enclosing the closed region.

**Figure 4:**
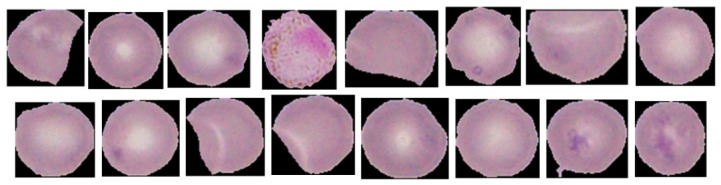
Segmented red blood cell images from the input image.

The final step in the segmentation process involves labelling each RBC and extracting its corresponding region of interest (ROI) from the original image, resulting in a set of cropped RBC images. This was achieved using the connected component labelling method, where similar pixels are grouped based on 8-neighborhood pixel connectivity. Once these groups, or blobs, were identified, each was assigned a unique label. The label data also contained the coordinates of the bounding box, which were then used to extract the original RBCs from the color images. Since other cells, such as platelets and white blood cells (WBCs) may also be segmented, area-based filtering is applied to retain only RBCs (DB1: 3500–5000 px, DB2: 2100–3000 px), by measuring the average pixel area for RBCs in the respective databases. This method removes the irrelevant cells based on size. The resulting RBCs are then cropped and resized to standardized color images for consistent analysis. The segmented cells from the acquired input image, using the proposed method as shown in Figure 3, are highlighted in Figure 4.

### 2.3. Binary Classification

#### 2.3.1. Data Preparation and Augmentation

All segmented RBC images were resized to 240 × 320 and 128 × 128 pixels to meet the input requirements of both pre-trained and custom CNN models, and normalized to speed up convergence. The dataset was split into training (70%), validation (10%), and testing (20%) sets, with 2422 images for training, 348 for validation, and 692 for testing—balanced across infected and normal RBCs. To improve generalization and prevent overfitting, image augmentation was applied to the training set, creating a more diverse dataset. Augmentation techniques included height/width shifts, rotation, zoom, shearing, and horizontal flips, performed dynamically during training. These augmented datasets were used across all models with adjusted final layers. Due to limited annotated medical data, such augmentation is critical for building robust generalized models for malaria diagnosis.

#### 2.3.2. A Customized Convolution Neural Network

**Figure 5:**
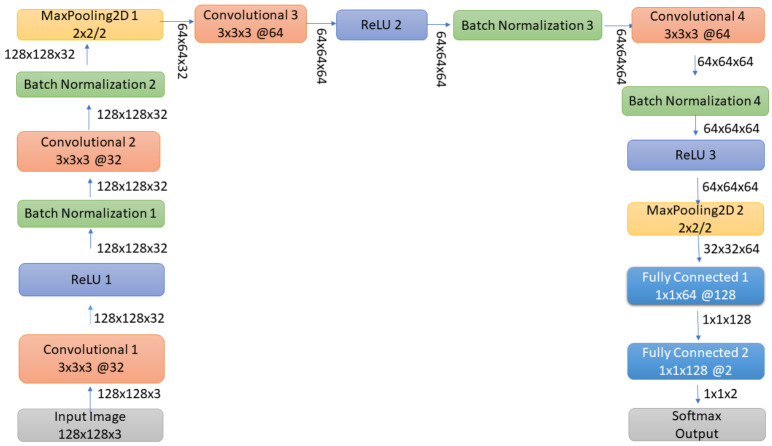
The architecture of the customized CNN model.

The architecture of the proposed CNN model for classifying segmented RBC images (128 × 128 × 3) is illustrated in Figure 5. The model consists of four convolutional layers and two fully connected layers. All convolutional layers utilize 3 × 3 filters with a stride of 1.

The first two convolutional layers each have 32 filters, while the third and fourth have 64 filters. To enhance learning efficiency, each convolutional layer is followed by a sandwich block comprising a *ReLU* activation function and batch normalization. Max-pooling layers with a 2 × 2 window and a stride of 2 are applied after the second and fourth convolutional layers to reduce spatial dimensions and retain important features. The output feature maps from the final convolutional layer are flattened and passed to the first fully connected (dense) layer with 128 neurons. Finally, the second fully connected layer serves as a *SoftMax* function that outputs the probability distribution across the target classes (infected vs. normal RBCs).

#### 2.3.3. Transfer Learning

Several CNN architectures utilize pre-trained weights from models trained on the ImageNet dataset for transfer learning [21], leading to substantial improvements in the performance of medical image analysis tasks. We assessed the performance of various pre-trained CNNs, including *VGG16*, *VGG19*, *DenseNet121*, and *InceptionV3*, in classifying parasitized and uninfected cells. To fine-tune these models for our dataset, which focuses on classifying infected or normal cells, additional customized fully connected layers were added. The additional fully connected layers utilize the rectified linear unit (*ReLU*) activation function and are defined as follows:

(2) ReLU=max(0,Z)

To predict cell image data, the final dense layer consists of two neurons, utilizing the *SoftMax* function defined as follows:

(3) $$s\left(Z_{i}\right)=\frac{e^{Z_{i}}}{\sum_{j=1}^{k}e^{Z_{i}}}$$

**Figure 6:**
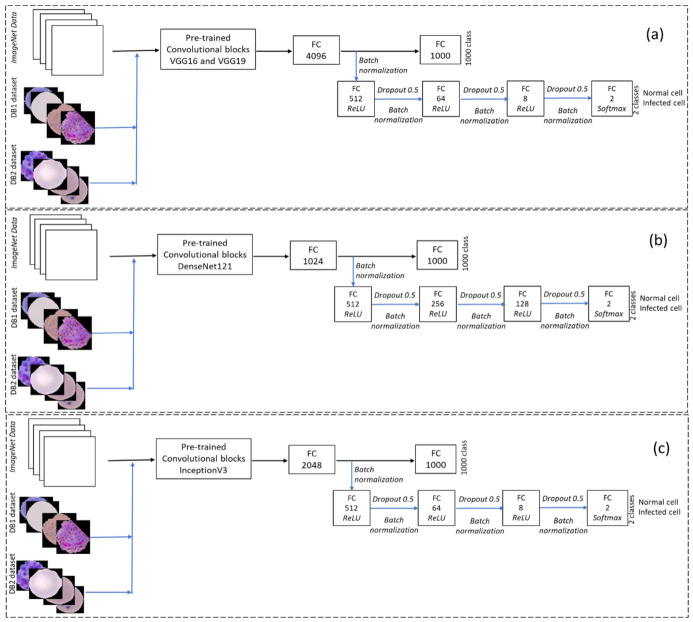
The proposed transfer learning scheme with different pre-trained classifiers: (**a**) *VGG16* and *VGG19*, (**b**) *DenseNet121*, and (**c**) *InceptionV3.*

Figure 6 illustrates the schematic transfer learning framework developed for binary classification of RBCs into infected and normal categories. Pre-trained models—*VGG16*, *VGG19*, *DenseNet121*, and *InceptionV3*—were originally trained for 1000 classes on the *ImageNet* dataset. In this transfer learning, all the pre-trained layers of these models were frozen, and new fully connected layers were added. The added layers included dropout and batch normalization, which are fine-tuned using our dataset to improve generalization and convergence. *VGG16* and *VGG19* are grouped within a single block due to their architectural similarity. Similarly, for *InceptionV3* and *DenseNet121* architectures, fully connected layers were added to update batch normalization, with these layers set as trainable to classify the two-class output.

The proposed methodology was trained on 2422 segmented RBCs, validated on 348 cell images and evaluated using 692 unseen RBC images from a separate test set. The RBC segmentation was performed using *MATLAB R2020a*, while model development and training were conducted in *Python* 3.10.12 using *Keras 2.9* with *TensorFlow 2.9* as the backend, accelerated by *CUDA 11* and an *NVIDIA RTX 3080* GPU. Pre-trained models from the *Keras 2.9* applications library were customized for the dataset. Additional libraries like *OpenCV 4.6*, *NumPy 1.23*, *Scikit-learn 1.1.3*, and *Matplotlib 3.6.2* supported image processing, evaluation, and visualization. The entire methodology was executed on a high-performance *Windows 11* Pro workstation with an *Intel Xeon W-1370P* CPU and 64 GB RAM.

#### 2.3.4. Performance Metrics

The proposed CNN architectures were evaluated using a comprehensive set of performance metrics to identify the best-performing model for RBC classification. A five-fold cross-validation approach was applied to reduce bias and enhance generalization, with training accuracy curves and confusion matrices used to monitor model behavior across folds. The optimal model was selected based on performance from the best fold. Further evaluation on a separate test set of 692 unseen images was conducted using classification reports, summarizing precision, recall, F1-score, accuracy, and weighted and macro accuracy. Additional metrics such as sensitivity, specificity, and the MCC were also calculated to provide a balanced and robust evaluation. MCC, in particular, offers a single, informative score for binary classification based on *TP*, *TN*, *FP*, and *FN*, and these values are utilized to calculate the performance metrics.

(4) $$Precision=\frac{TP}{TP+FP}$$

(5) $$Recall=\frac{TP}{TP+FN}$$

(6) $$F1\;score=\frac{2\times Precision\times Recall}{Precision+Recall}$$

(7) $$Accuracy=\frac{\sum_{c=1}^{l}{TP}_{c}}{N}$$

(8) $$Macro\;Average=\left\{\begin{array}{c} \frac{\sum_{c=1}^{l}Precision}{l}\\ \frac{\sum_{c=1}^{l}Recall}{l}\\ \frac{\sum_{c=1}^{l}F1\;score}{l} \end{array}\right.$$

(9) $$Weighted\;Average=\sum_{c=1}^{l}w_{c}\times \left\{\begin{array}{c} \frac{\sum_{c=1}^{l}Precision}{l}\\ \frac{\sum_{c=1}^{l}Recall}{l}\\ \frac{\sum_{c=1}^{l}F1\;score}{l} \end{array}\right.$$

(10) $$Specificity=\frac{TN}{TN+FP}$$

(11) $$Sensitivity=\frac{TP}{TP+FN}$$

(12) $$MCC=\frac{\left(TP\times TN\right)-\left(FP\times FN\right)}{\sqrt{\left(\left(TP+FP\right)\left(TP+FN\right)\left(TN+FP\right)\left(TN+FN\right)\right)}}$$

#### 2.3.5. AI-Based Web Application for Cell Classification

**Figure 7:**
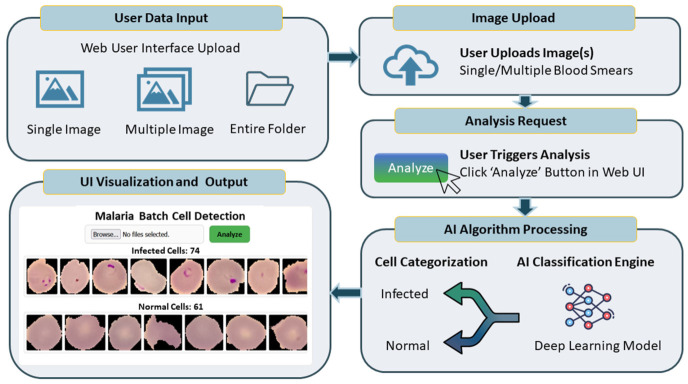
AI-enabled cells classification web-based application.

The end-to-end workflow of the proposed AI-enabled malaria cells classification application is depicted in Figure 7. The application is developed using a stack of Python, HTML, and JavaScript; the system leverages a Flask-based backend server, currently validated in a local host environment. The process is structured and involves the following steps:

Data Acquisition: Users initiate the workflow by uploading images via the web interface, which supports single-image, multi-image, or batch folder uploads.

Analysis Initiation: Upon clicking the “Analyze” button, the interface triggers a request to the backend AI engine.

AI Classification: The server-side engine processes the raw data to categorize cells into “Infected” or “Normal” classes using the optimized deep learning model.

Data Streaming and Visualization: The results are transmitted back to the client via data streaming, accompanied by metadata containing the total cell counts for both classes.

User Interface (UI) Rendering: The frontend visualizes the categorized results in a scrollable output area, allowing the user to review the entire set of classified cell images.

## 3. Results and Discussion

To recognize RBCs, they were first segmented using the image processing approach described in Section 2. The segmentation time for one image from dataset DB1 was 4.195 s, while for an image from DB2, it was 0.509 s. Subsequently, a customized CNN model and *ImageNet* pre-trained models with additional modified fully connected layers were trained using these augmented samples. The training, validation, and test datasets were strictly separated to prevent any leakage of staining variations or other artifacts. All image datasets were normalized in the range [0, 1].

**Figure 8:**
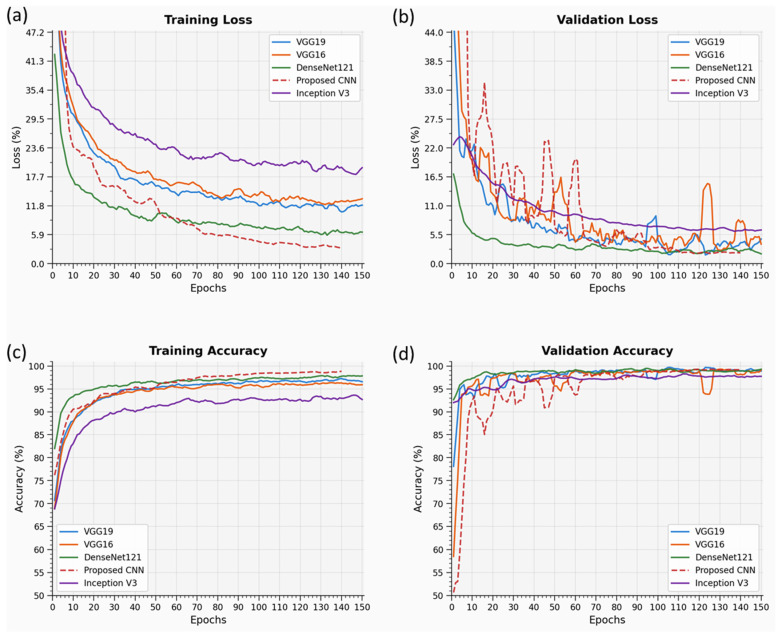
VGG16, VGG19, DenseNet121, Inception V3 and a proposed CNN: (**a**) training loss, (**b**) validation loss, (**c**) training accuracy, and (**d**) validation accuracy for all models.

The networks were trained over 75, 150, 225, and 300 epochs, with a batch size of 32. Hyperparameter optimization for all CNN models was performed using a random grid search within the range [1 × 10^−7^, 5 × 10^−2^], employing *Adam*, *RMSprop*, and *SGD* optimizers. The *Adam* optimizer, with a learning rate of 1 × 10^−4^, was ultimately selected for minimizing cross-entropy loss and producing probability-based predictions. The custom CNN attains optimal convergence through (a) optimization of its architecture and hyperparameters, (b) the use of batch normalization for implicit regularization, and (c) improved generalization achieved by applying aggressive dropout to both convolutional and dense layers. Optimal convergence for all models was achieved at 150 epochs. The best-performing architectures were evaluated through training accuracy curves and confusion matrices using five-fold cross-validation. The performance of these models during training in terms of training accuracies and losses for 150 epochs is presented in Figure 8.

A comparative performance analysis of different architectures is summarized in Table 3. Among all tested models, *VGG* networks outperformed others in terms of accuracy across all epochs, with *VGG19* emerging as the top performer based on training time compared to *VGG16*. *VGG19* was selected for further comparative analysis across all five networks.

**Figure 9:**
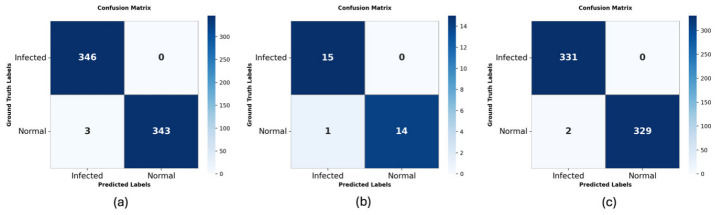
Confusion matrix for the pre-trained *VGG19* network with additional fully connected layers applied to (**a**) test data, (**b**) DB1, and (**c**) DB2.

The test accuracy of *VGG19* on the total test dataset is compared with different models in Table 3. The individual cell classification confusion matrix is depicted in Figure 9a, and their performance classification report that includes the precision, recall, F1-score and support is shown in Table 4. The sensitivity and specificity for the *VGG19* model on combined data is 99.42.

The performance classification for the test dataset DB1 is reported. The DB1 individual cell classification performance is detailed in Table 5, and the confusion matrix in Figure 9b. Sensitivity and specificity for the *VGG19* model on the DB1 dataset is 96.66.

Table 6 presents the performance of the cell classification of the selected model on the dataset DB2. Figure 9c shows the confusion matrix. Sensitivity and specificity for the VGG19 model on the DB2 dataset is 99.54.

For 150 epochs, the *VGG19* transfer learning model needed a training time of 86.02 min for two-class cell classification. The *VGG19* model’s weight size was 126.87 MB, and the total model size was 321.77 MB. Despite the model’s larger size and weight, the *VGG19* network outperformed other models for malaria cell classification. The comparative assessment demonstrates that the *VGG19* pre-trained model, as a transfer learning, efficiently classifies malaria cells, achieving accuracy comparable to state-of-the-art methods, as shown in Table 7.

## 4. Limitations

The present work should be interpreted as a proof-of-concept study focused on image-level classification of segmented red blood cells as infected or uninfected using computer vision and CNN architectures. There is no direct comparison with conventional reference malaria diagnostics such as microscopy, rapid diagnostic tests, or molecular assays as it was not designed as a prospective diagnostic accuracy study, which would include indices such as sensitivity, specificity, positive predictive value, and negative predictive value. The findings, therefore, primarily demonstrate the technical feasibility and high classification performance of the tested approaches, while future studies should address external validation, comparison with existing diagnostic tests, and evaluation in real-world settings.

## 5. Conclusions

Computer-aided image analysis using DL, particularly CNNs, offers significant advantages in medical diagnostics by enabling end-to-end feature extraction and classification directly from raw data. Our findings are broadly consistent with prior studies showing strong performance of deep learning and transfer learning models for malaria image classification. However, direct comparisons across studies remain difficult because of differences in image datasets, preprocessing strategies, class balance, and evaluation protocols. This study implemented both customized CNN models and four pre-trained architectures, demonstrating high predictive performance with efficient training times. Among them, VGG19 achieved the best results, with 99.57% test accuracy for a wide variation of blood smear cell datasets combining state-of-the-art and lab-acquired data. To enhance usability, a web-based application was developed, allowing real-time RBC classification. The deployed model functions effectively as a triage tool, showing strong generalization, minimal overfitting, and potential for clinical use in detecting parasitized and normal cells in blood smear images. Thus, the proposed model may be used in the future as a supportive computational approach for image-based screening, alongside standard diagnostic modalities.

## Figures and Tables

**Table 1:** Deep learning architectural and accuracy comparison.

Study	Architecture and Paradigm	Dataset	Task	Reported Performance Metrics
ROENet (2022) [27]	Pre-trained ResNet-18 feature extractor mapped to an ensemble of three randomized neural networks (RVFL, SNN, ELM).	Public NIH Dataset 27,558 single-cell cropped image patches. Balanced classes.	Binary patch classification (parasitized vs. uninfected).	Accuracy: 95.73% Sensitivity: 94.79% Specificity: 96.68% F1-Score: 95.69%
Chaudhry et al. (Springer, 2024) [28]	Custom Ultra-Lightweight CNN framework using Global Average Pooling (GAP). Optimized to contain <0.4 M trainable parameters.	Evaluated on four public datasets (MP-IDB, MP-IDB2, IML_Malaria, Malaria-Detection 2019	Multi-class classification (parasite species typing and four life-cycle stages).	Multi-class Accuracy: MP-IDB: 99.0% MP-IDB2: 96.0% IML_Malaria: 92.0% MD-2019: 82.0%
Wang et al. (Oxford, 2023) [29]	YOLOv7 single-stage object detection framework with structural reparameterization and Path Aggregation Network (PAN).	Multicenter clinical dataset. 12,708 high-resolution full thin-smear images	Simultaneous end-to-end object detection and species/stage classification.	Mean Average Precision: mAP@0.5: 0.902 RBC Recall: 96.0% RBC Precision: 94.9%
Proposed Study	Two-step cell segmentation with transfer learning	Acquired dataset and public dataset	Cell segmentation and binary classification (infected vs. non infected)	99.57%

**Table 2:** DB1 and DB2 dataset statistics.

Image Dataset	Whole Image	Individual Cell Image							
		PFR	PFT	PFS	PFG	PVR	PVS	PVG	NOR
DB1	581	647	256	65	289	196	50	148	1648
DB2	35	29	13	3	6	10	4	7	72
Total	616	676	269	68	295	206	54	155	1720

**Table 3:** Performance metrics for transfer learning and proposed CNN models.

Model	Accuracy	Sensitivity	Specificity	F1-Score	MCC
VGG19	99.57	99.57	99.57	99.57	99.13
VGG16	99.28	99.28	99.28	99.28	98.56
DenseNet121	98.69	98.69	98.69	98.69	97.43
Proposed CNN	99.14	99.43	98.85	99.14	97.95
InceptionV3	97.10	97.10	97.10	97.10	94.24

**Table 4:** Classification report for the *VGG19* network with additional fully connected layers applied to the test data.

Class	Precision	Recall	F1-Score	Support	95% CI
Infected	99.14	100.00	99.57	346	Precision: 97.51–99.70; Recall: 98.90–100.00
Normal	100.00	99.13	99.56	346	Precision: 98.89–100.00; Recall: 97.47–99.69
**Accuracy**			**99.57**	692	98.97–99.89
Macro accuracy	99.57	99.57	99.57	692	98.97–99.89
Weighted accuracy	99.57	99.57	99.57	692	98.97–99.89

**Table 5:** Classification report for the *VGG19* network with additional fully connected layers on DB1 dataset.

Class	Precision	Recall	F1-Score	Support	95% CI
Infected	93.75	100.00	96.77	15	Precision: 71.67–98.89; Recall: 79.61–100.00
Normal	100.00	93.33	96.55	15	Precision: 78.47–100.00; Recall: 70.18–98.81
**Accuracy**			**96.67**	30	83.33–99.41
Macro accuracy	96.88	96.67	96.66	30	83.33–99.41
Weighted accuracy	96.88	96.67	96.66	30	83.33–99.41

**Table 6:** Classification report for the VGG19 network with additional fully connected layers on the DB2 dataset.

Class	Precision	Recall	F1-Score	Support	95% CI
Infected	99.40	100.00	99.70	331	Precision: 97.84–99.84; Recall: 98.85–100.00
Normal	100.00	99.49	99.70	331	Precision: 98.85–100.00; Recall: 97.82–99.83
**Accuracy**			**99.70**	662	98.91–99.92
Macro accuracy	99.70	99.70	99.70	662	98.91–99.92
Weighted accuracy	99.70	99.70	99.70	662	98.91–99.92

**Table 7:** Comparison of classification accuracy with state-of-the-art methods.

Ref.	Dataset and Size	Methods/Features	Classifiers	Accuracy
Kanojia et al. [31]	CDC Malaria dataset (134)	Ostu’s global thresholding, Marker-controlled watershed segmentation, Shape Descriptor Features, (GLCM)	Levenberg – Marquardt Back Propagation Neural Network	72%
Pan et al. [32]	PEIR-VM (4800 segmented Single Cell images)	Data augmentation and Transfer learning	*LeNet-5* architecture	79%
Rajaraman S. et al. [25]	NIH dataset (27,558 segmented single cell images)	Transfer learning	Pre-trained models as optimal feature extractors and customized CNN architecture were compared	95.9%
A V, B RK et al. [33]	Self-collected (2550 cropped single cell images)	Transfer learning	Unification of *VGG19* and SVM	93.13%
Liang Z. et al. [34]	NIH dataset (27,558 segmented single cell images)	Transfer learning	Transfer learning and customized CNN models were compared	97.37%
Rajasekar S S. et al. [35]	NIH dataset (27,558 segmented single cell images)	Graph Attention Networks (GATs) with Yen’s Iterative Thresholding (YIT)	Kernel Spectral Clustering (KSC)	96.00%
Madhu et al. (2023) [36]	NIH dataset (27,558 segmented single cell images)	*Inception v3*-based feature extraction	Capsule neural network	99.35%
Özdemir et al.(2025) [37]	Kagle dataset (27,560 segmented single cell images)	Feature extraction using *ViT* and *EfficientNet*	*CatBoost*, *XGBoost*, and Logistic Regression	95.61%
Khan et al. (2025) [38]	NIH dataset (27,558 segmented single cell images)	Fractional order optimization	*SoftMax*	95.60%
Proposed Transfer Learning Approach	CDC Malaria dataset and self-collected (3498 segmented single cell images)	Data augmentation and transfer learning	*VGG19* architecture followed by fully connected dense layers for two classes	Test data Images: 692 images *VGG19*: 99.57%

## Data Availability

The data used in this manuscript will be available on request to the corresponding author.
